# Gingival Manifestations in Oral Chronic Autoimmune Bullous Diseases: A Retrospective Study

**DOI:** 10.3390/medicina60010167

**Published:** 2024-01-17

**Authors:** Ioanina Parlatescu, Serban Tovaru, Cristina Tofan, Paula Perlea, Elena Milanesi, Maria Dobre, Laurenta Lelia Mihai

**Affiliations:** 1 Faculty of Dentistry, Carol Davila University of Medicine and Pharmacy, 020021 Bucharest, Romania; ioanina.parlatescu@umfcd.ro (I.P.); serban.tovaru@gmail.com (S.T.); paula.perlea@umfcd.ro (P.P.); 2 Private Dental Practice, Dimitrie Cantemir Street, no 11, bl. 8, ap. 46, 040233 Bucharest, Romania; 3 Faculty of Medicine, Carol Davila University of Medicine and Pharmacy, 020021 Bucharest, Romania; elena.milanesi@ivb.ro; 4 Victor Babeș National Institute of Pathology, 050096 Bucharest, Romania; maria.dobre@ivb.ro; 5 Faculty of Dental Medicine, “Titu Maiorescu” University of Bucharest, 031593 Bucharest, Romania; lelia_mihai2000@yahoo.com

**Keywords:** bullous lesions, gingival diseases, mucous membrane pemphigoid, oral mucosa, pemphigus

## Abstract

*Background and Objectives*: Desquamative gingivitis (DG) is a clinical term indicating “peeling gums” and is associated with different oral manifestations. In this study, we aimed to assess the association between DG and autoimmune blistering mucocutaneous diseases (ABMD) with oral manifestations. *Materials and Methods*: A retrospective study including 88 patients diagnosed between 1998 and 2019 with ABMD (intraepithelial and subepithelial autoimmune blistering diseases) was performed at the Oral Medicine Department, Faculty of Dentistry, “Carol Davila” University of Medicine and Pharmacy in Bucharest. For each patient, the sociodemographic and anamnestic data, as well as clinical features of oral lesions (location), histological evaluation, and direct immunofluorescence data were collected. *Results*: Most of the patients involved in the study were female (78.4%). In total, 34 patients (38.63%) were diagnosed with subepithelial autoimmune diseases (SAD) and 54 (61.36%) had intraepithelial autoimmune diseases (IAD). Differences in the anatomic distribution of oral involvement were found between SAD and IAD. The presence of DG was significantly more common in patients with SAD compared to those with a diagnosis of IAD. *Conclusions*: Specific anatomical locations of the oral lesions are significantly associated with different subtypes of ABMD, with gingiva and hard palate mucosa being more involved in SAD and the soft palate and buccal mucosa in IAD. Desquamative gingivitis is a clinical sign that raises diagnostic challenges for several conditions in oral medicine.

## 1. Introduction

Desquamative gingivitis (DG) is a clinical term used for non-plaque associated gingival lesions identified as erythema, desquamation, erosions, and blisters. This clinical manifestation mainly affects middle-aged and older adults, with a 4:1 female to male ratio [1]. DG is a sign of gingival involvement in several disorders, causing a range of severe symptoms, including discomfort and pain when eating, drinking, or brushing teeth. Its presence can be solitary or in combination with other cutaneous or mucosal lesions. The etiology of DG comprises several mucocutaneous dermatoses (lichen planus, autoimmune bullous diseases), as well as hormone imbalance, contact allergic reactions, and chemical and electrical burns [2,3]. In oral chronic ulcers, the presence of the Nikolsky sign (represented by horizontal, tangential pressure on the healthy mucosa adjuvant to a lesion, leading to blisters enlarging or disruption) is a viable test clinically highly suggestive of the presence of oral bullous disease [4]. The clinical criteria for the diagnosis of DG have been firstly established by Nisengard and Levine [5] in 1995. These criteria included the presence of erythema non-plaque induced and desquamation on the gingiva, together with additional oral or extraoral lesions and sensations of sore mouth, primarily due to spicy food. Later, in 2018, the World Workshop on the Classification of Periodontal and Peri-implant Diseases and Conditions [6,7] had included in the non-dental biofilm (non-plaque) induced gingival diseases and conditions in subheading 3: the inflammatory and immune conditions and lesions with the clinical features of desquamative gingivitis (erythematous, vesiculo-bullous, erosive lesions). Within this classification, the term “DG” is only used for pemphigus vulgaris and pemphigoid, even if lichen planus and hypersensitivity reactions overlap with certain characteristics of DG.

Since DG is a clinical sign encountered in several conditions, the final diagnosis requires additional investigations such as histopathological evaluation and immunofluorescence in the case of a positive Nikolsky sign [1]. Furthermore, the presence of persistent ulcerative bullous lesions on the oral mucosa, particularly at the gingival level, raises the suspicion of autoimmune mucocutaneous bullous diseases.

Based on the level of blister formation, the autoimmune mucocutaneous bullous diseases (ABMD) are divided into two main categories: intraepithelial/intraepidermal autoimmune diseases (IAD) and subepithelial autoimmune diseases (SAD). 

IAD is triggered by antibody response against desmosomes, while in SAD, the immune response is directed against the structural components of the basement membrane complex and hemidesmosomes [8]. The intraepithelial category of autoimmune mucocutaneous bullous diseases, also entitled the pemphigus group, comprises the following diseases: pemphigus vulgaris, pemphigus foliaceus, pemphigus vegetans, pemphigus herpetiformis, IgA pemphigus, and IgG/IgA pemphigus. On the other hand, the subepithelial category, termed the pemphigoid group, include bullous pemphigoid, mucous membrane pemphigoid, pemphigoid gestationis, anti-p200 pemphigoid, lichen planus pemphigoides, epidermolysis bullosa acquisita, and linear immunoglobulin A bullous dermatosis. Autoimmune mucocutaneous bullous diseases often occur on the oral mucosa and lastly spread to the skin and other mucosa (conjunctiva, genital, nose, esophagus) [9]. In certain cases, the oral mucosa is the only area affected.

Since the clinical oral features of autoimmune mucocutaneous bullous diseases are polymorphic and show similarities, the diagnosis is made by standard investigations such as histopathologic evaluation, direct immunofluorescence, and immunoserologic tests [10,11]. The therapeutic approach of DG is challenging mainly due to its different etiology. Sometimes, a multidisciplinary context (Dentistry, Dermatology, Allergology, etc.) is required to assess the general health status of patients and to avoid drug interactions and side effects.

The presence of gingival lesions and the associated pain and discomfort during tooth brushing, or even minor trauma to the lesional areas, underline the idea that patients with DG have difficulty maintaining adequate oral hygiene. As a result, it is easy to get caught in a vicious cycle of pain/discomfort or even bleeding when brushing, poor hygiene, bacterial plaque formation, increased local inflammation, and exacerbation of the underlying inflammatory autoimmune disease. Research data on the relationship between dental hygiene and DG in autoimmune diseases are scarce, with only a review on the topic, indicating that effective management of bacterial plaque can reduce symptoms as well as the severity of lesions [12].

In the pemphigus group, the suprabasal cleft and acantholysis that cause blisters are due to IgG autoantibodies that, binding desmoglein (Dsg) (1, 3, or both), induce the weakening and the disruption of the desmosomes that connect the epithelial cells. Desmoglein 3 autoantibodies are expressed in the mucosal dominant phenotype of pemphigus (pemphigus vulgaris) and desmoglein 1 autoantibodies are present in cutaneous pemphigus [10]. Pemphigus vulgaris mainly affects adults between the fourth and sixth decade of life and has a slight female predominance [13,14]. In 50% of cases of PV, oral lesions are usually the first sign, occur before the cutaneous lesions, and are difficult to resolve with treatment [15]. Paraneoplastic pemphigus is a rare and potentially life-threatening autoimmune disease associated with an underlying neoplasm (lymphoma and hematologic malignancies) [16]. This form of pemphigus is associated with autoantibodies directed against the plakin family (envoplakin, periplakin, the desmoplakins) as well as against plectin, 230 BP antigen, plakophilin 3, desmocollin 1, and desmocollin 3, Dsg 1 and Dsg3 [16].

The autoimmune response in the pemphigoid group is focused on specific antigens of the dermal-epidermal/stromal-epithelial junction: Bullous pemphigoid is associated with antibodies against antigen 180 bullous pemphigoid (BP180), 230 bullous pemphigoid (BP230), and mucous membrane pemphigoid (MMP), which involves several autoantigens such as BP180, laminin-332, α6β4 integrin and type VII collagen. Autoantibodies against type VII collagen are associated with epidermolysis bullosa acquisita [11]. As MMP in which more than one mucosa is affected shows distinct features, the European guidelines for MMP diagnosis and management [17] have recognized this condition as a “disease phenotype”. It has been suggested to adopt the terms ocular MMP and oral MMP in cases where only one mucosal site is involved, either ocular or oral. 

In this article, we report the results of a retrospective study conducted on Romanian patients to assess a possible association between DG and oral intraepithelial and subepithelial chronic autoimmune diseases.

## 2. Materials and Methods

### 2.1. Patients and Inclusion/Exclusion Criteria

We performed a retrospective single-center descriptive study on 88 patients diagnosed with oral autoimmune blistering diseases at the Oral Medicine Department, Faculty of Dentistry, “Carol Davila” University of Medicine and Pharmacy in Bucharest from 1998 to 2019. The study was developed according to the STROBE checklist included as Supplementary Material. The diagnosis of the autoimmune blistering mucocutaneous disease (ABMD) was established by clinical signs, histological findings, and direct immunofluorescence results [10,18]. 

The data collected from the medical records covered the inclusion and exclusion criteria for the current study, which are presented in Table 1.

Other investigations recommended as diagnostic tools and disease activity monitoring, such as indirect immune fluorescence microscopy to detect serum autoantibodies and enzyme-linked immunosorbent assay (ELISA Test) [8,9,18], were not carried out in our department. All of the ABMD patients involved in the study received a diagnosis of intraepithelial or subepithelial dermatosis by a dermatologist, which also prescribed a therapeutic regimen.

According to this diagnosis, we stratified our cohort into two groups: patients with intraepithelial autoimmune dermatoses (IAD) and subepithelial autoimmune dermatoses (SAD). The first group of diseases included the diagnosis of pemphigus vulgaris and paraneoplastic pemphigus, which are dermatoses with oral involvement [19]. The second group of diseases included the diagnosis of mucous membranous pemphigoid, bullous pemphigoid, and other subepithelial autoimmune dermatoses.

Data regarding the sociodemographic data (age, gender, smoker status, environment, and level of education), other associated diseases, the duration of disease before a first visit, and descriptive analysis of the oral lesions were retrieved from the medical files. The WHO’s guidelines were followed in classifying the oral mucosa’s topography [20]. For each patient, a tissue sample was obtained from the perilesional region, and the diagnosis was confirmed by direct immunofluorescence and histological analysis.

The study was carried out following the declaration of Helsinki and approved by the “Carol Davila” University of Medicine and Pharmacy Ethics Committee (number 164/2018).

### 2.2. Statistical Analysis

Differences in continuous variables (reported as mean ± SD) were tested using Student’s *t*-test, whereas categorical variables (reported as absolute values and percentages) were tested by the Chi-square test when the number of patients in a subgroup was ≥5, and the Fisher exact test was used when the number was less than 5 in any cell. The statistical analysis was performed using the Statistical Package for the Social Sciences (SPSS version 18.0). 

## 3. Results

In total, 88 patients diagnosed with oral manifestations of autoimmune blistering mucocutaneous disease were included in this analysis. All of the patients were referred to the Oral Medicine Department for diagnosis of the oral lesions. The mean age of the patients was 59.18 ± 14.56 years (range 23–86 years). Most of the recruited patients were female in the ratio of 6:1.65 (female: male), accounting for 78.4% of the patients. Overall, 65 (73.86%) patients were non-smokers, and 74 (84%) lived in an urban area. In total, 34 patients were diagnosed with subepithelial autoimmune diseases (SAD) (38.63%) and 54 had intraepithelial autoimmune diseases (IAD) (61.36%) (Table 2).

The mean of the self-reported lesion duration before the first visit was statistically significantly lower in SAD patients (6.07 months) compared to individuals with IAD (20.32 months). 

Regarding the associated medical comorbidities, 29.41% of SAD patients and 7.40% of IAD patients reported being previously diagnosed with other autoimmune diseases (autoimmune thyroiditis, atopic dermatitis, rheumatoid polyarthritis, immune thrombocytopenic purpura, etc.). 

The direct immunofluorescence studies revealed that IgG was present in most of the cases (85.29% in SAD and 64.81% in IAD).

In Table 3 is the reported frequency of lesions by anatomical distribution. 

**Figure 1:**
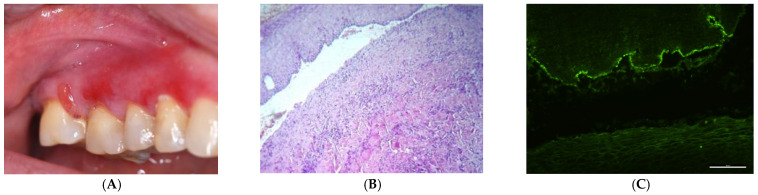
(**A**) Desquamative gingivitis with erythema and bullae in subepithelial autoimmune disease; (**B**) Histopathology image with epithelium detached from the connective underlying tissue and inflammatory infiltrate with neutrophils, lymphocytes, and plasma cells. (Hematoxylin and eosin, ×100); (**C**) Direct immunofluorescence examination shows linear and granular deposits of IgG along the basement membrane zone.

**Figure 2:**
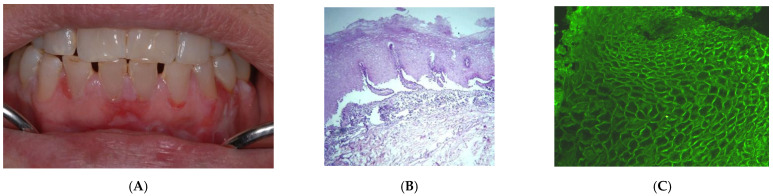
(**A**) Desquamative gingivitis with ulcers and desquamation in intrapithelial autoimmune disease; (**B**) Formation of suprabasal bullae (Hematoxylin and eosin, ×100); (**C**) Direct immunofluorescence examination shows intercellular fluorescence dispersed throughout the basal zone at a moderate intensity for IgG.

Desquamative gingivitis was present in 42 patients (47.72%) diagnosed with ABMD, in 27 (79.41%) patients diagnosed with SAD and in 15 (27.77%) patients with IAD. Moreover, it was the sole oral manifestation observed in 12 cases (5.55%) of IAD patients and 9 (26.47%) SAD patients. Figure 1 and Figure 2 present gingival lesions, histopathology, and direct immunofluorescence images of patients with SAD (Figure 1A–C) and IAD (Figure 2A–C).

For the SAD patients, the second involved oral mucosa area was the buccal mucosa (41.17%) followed by the soft palate (32.35%) and hard palate (17.65%). The buccal mucosa involvement was unilateral in all SAD patients. In IAD patients, the buccal mucosa (83.33%) was most frequently affected by lesions, followed by the soft palate (46.29%), and tonsillar pillar equal to the gingiva (27.77%). 

In seven (12.96%) of the IAD patients and eleven (32.35%) of the SAD patients, the oral lesions involved a single region. 

Extraoral involvement at the first visit was reported by six SAD patients (three patients with skin lesions and three patients with other mucosa involved) and fourteen IAD patients (eight patients with skin lesions and six patients with other mucosa lesions).

## 4. Discussion

The present retrospective study reports data on a cohort of 88 patients with oral manifestations of autoimmune blistering disease, collected in Romania between 1998 and 2019. The patients were referred mainly by dentists, general practitioners, ENT doctors, or other medical specialists for the diagnosis of chronic oral mucosal lesions. The final diagnosis and the therapeutic regimen have been established by dermatologists. We compared the oral clinical aspects between intra- and sub-epithelial blistering autoimmune diseases, finding differences in the anatomic distribution of oral involvement, and that the presence of DG was significantly more common in patients with SAD. 

The IAD group with oral involvement includes pemphigus vulgaris (PV) and paraneoplastic pemphigus. Since PV is the most frequent form of intraepithelial autoimmune diseases, and the oral mucosa is damaged in up to 70% of patients [19], most of the studies in different populations have focused on oral symptoms and lesions of PV [19,21,22,23].

Regarding SAD, the mucous membranous pemphigoid (MMP) is the most studied disease in the subepithelial autoimmune blistering group with oral manifestations. In 85% of patients, the oral mucosa is the site of onset [17]. The evolution is characterized by episodes of relapses and remissions [17]. The European consensus on bullous pemphigoid (BP) [24] stated that lesions of the oral mucosa are uncommon; nonetheless, different studies report varying percentages of oral involvement, ranging from 11.4% [25] to 27% [26] or even 40% [27] in different populations.

Our study results are in line with previous findings from other research groups which identified a high frequency of females affected by oral PV [1,23,27,28] and SAD (MMP and BP) [22,29,30]. The European criteria for MMP [17] suggest that MMP is diagnosed between 60 and 80 years of age, which is consistent with the mean age of patients with SAD in our cohort—66 years. The MMP, formerly known as cicatricial pemphigoid, is a collection of disorders and is referred to as a “disease phenotype.” When there is only oral involvement, the expression oral MMP should be used. Rarely does scarring and fibrosis aggravate oral lesions.

Since oral lesions are often not properly recognized by medical professionals, and most patients prefer to wait for spontaneous healing rather than consult a doctor, the diagnosis of these diseases is usually delayed [31,32]. In our study, we observed a statistically significant difference in the mean of the self-reported duration of oral lesions between the IAD and SAD groups. Accordingly, patients with SAD diseases presented after a mean period of 20.32 months, while those with IAD diseases presented after a mean period of 6.07 months. Daltaban and collaborators [28] reported in patients with PV and oral involvement a diagnostic delay period of 6 months, which was statistically correlated with the number of previous consultations. A proper early diagnosis of IAD, as well as the identification of a treatment, reduces the potential that lesions develop on the skin and other mucosae [28].

When we compared the clinical lesions between the SAD and IAD groups, we found significant differences regarding the anatomic distribution of oral involvement. In particular, in SAD patients, the gingiva and the hard palate were significantly more frequently involved with oral lesions, whereas in IAD patients the most affected anatomical regions were buccal mucosa and the soft palate. In contrast with our results, Laskaris et al. [29] reported the soft palate as the most common oral site involved in 157 patients diagnosed with PV.

Desquamative gingivitis is a clinical sign presenting with erythema, erosions, bullae, and ulcers. It is appreciated as a non-specific feature of a large variety of diseases such as oral lichen planus, autoimmune blistering diseases (PV, MMP, epidermolysis bullosa, epidermolysis bullosa acquisita, linear IgA disease), lupus erythematosus, graft-versus-host disease, chronic ulcerative stomatitis, erythema multiforme, adverse drug reactions, and orofacial granulomatosis [33]. In oral autoimmune diseases, the presence of desquamative gingivitis is associated with a considerably longer delay of diagnosis than in patients with mucosal ulcers [31].

As in our cohort’s gingival involvement of ABMD showed statistically significant differences, we compared our findings with previous research on DG. There is a large range of variability in the frequency of DG reported for autoimmune diseases with oral manifestations. Therefore, 70% of the cases with the diagnosis of oral lichen planus, followed by 14% MMP and 13% PV, were reported by Leao et al. [34] in their analysis of 187 DG patients in the UK. After analyzing 382 DG cases of Italian patients, Arduino et al. [35] concluded that the most common diagnosis was oral lichen planus, followed by MMP, PV, and epidermolysis bullosa acquisita. Sklavounou and Laskaris’s findings [36], however, disagree in that they show DG to be more common in MMP patients than in oral lichen planus patients. In this study, we included only patients with ABMD and excluded the patients diagnosed with oral lichen planus.

In our cohort, desquamative gingivitis has been observed in almost half of the ABMD patients, and in 12 patients (13.63%) it was the unique oral lesional site. The presence of DG was significantly more frequent in patients with SAD than in those belonging to the IAD group (79.41% vs. 27.22%). When comparing gingival lesions in ABMD patients, there is no major regional difference; comparable results have been found in the US population [22] and the Greek population [29]. Accordingly, Laskaris et al. [29] reported gingival involvement in 63.6% of cicatricial pemphigoid cases, 16% of bullous pemphigoid cases, and in 25.2% of pemphigus vulgaris patients. Sultan et al. [22] observed that desquamative gingivitis was present in 84% of pemphigoid patients and in 26% of pemphigus cases. Although according to Jaschot et al., analyzing the periodontal status in oral pemphigus and pemphigoid identified conflicting findings between studies, the authors showed that individuals with oral pemphigus and pemphigoid are more prone to periodontitis [37].

This study has potential limitations. One is the limited sample size mainly due to the patients’ referral. The primary focus of our university’s oral medicine clinic is the patients with oral mucosa disorders. However, the general practitioners and dermatological clinics, where ABMD patients typically seek care initially, are not accessible to us. Another limitation of the present study is that we did not perform a circulating serum autoantibodies evaluation or ELISA test, and we referred the patients to dermatology for treatment and follow-up.

Our perspective for clinical research on DG includes broadening the study cohort to dermatology hospitals, evaluating lesions following professional teeth cleaning in the presence and absence of treatment, as well as correlation of the DG occurrence with the level of circulating autoantibodies in patients diagnosed and treated for ABMD. Additionally, we plan to assess the effectiveness of local therapy in situations including distinct gingival involvement. Furthermore, a comparison of the patient’s susceptibility to periodontal disease, responsiveness to treatment, and course of the disease can show which variables are predictive of maintaining the structural integrity of the dental arches and reducing symptoms. More research is required to determine whether the existence of desquamative gingivitis alters dentists’ existing treatment protocols, and to better understand the constraints and particulars of these instances.

Because in some cases the manifestations of these autoimmune blistering mucocutaneous diseases involve the oral cavity, the patients typically consult dentists, who can aid in the diagnosing process and ultimately contribute to the best course of therapy. In the presence of gingival lesions, the diagnosis sometimes raises difficulties. The bacterial dental plaque added to the gingival lesions from autoimmune diseases aggravates the inflammation, altering their clinical appearance, and making identification more challenging. Thus, appropriate oral hygiene is one important matter for these patients.

The key to diagnosing these individuals appropriately is knowing the clinical characteristics of the oral lesions and performing a thorough and meticulous examination of the oral mucosa. Their detailed diagnosis and therapeutical management impose a collaborative approach between dermatologists, oral medicine specialists, and dentists.

## 5. Conclusions

Distinct anatomical locations of the oral lesions are significantly involved in autoimmune chronic blistering diseases; the gingiva and hard palate mucosa in subepithelial diseases and the soft palate and buccal mucosa in intraepithelial diseases. The gingival involvement has the clinical aspect of desquamative gingivitis. This non-specific clinical sign raises diagnostic challenges for several conditions in oral medicine and dentistry, mainly when it is a unique manifestation. Histopathological and immunological criteria reveal the diagnosis. In rare cases, when desquamative gingivitis is the only clinical sign, subepithelial autoimmune disease is more likely to be the cause.

## Figures and Tables

**Table 1:** The inclusion and exclusion criteria for the selection of patients.

Inclusion Criteria	Exclusion Criteria
A detailed anamnesis regarding the symptoms and a chronic onset of lesions, Presence of clinical lesions (eythema/erosions/ulcers/blisters) with a positive Nikolsky sign, Histopathological findings suggestive for acantholisis or subepithelial cleavage, Direct immunofluorescence results, Informed written consent.	Incomplete clinical data, Previous diagnostic of cutaneous autoimmune bullous diseases, Histopathology findings suggestive of other oral diseases (oral lichen planus), Lack of direct immunofluorescence results.

**Table 2:** Patients baseline characteristics.

	Subepithelial Autoimmune Diseases Group (%)	Intraepithelial Autoimmune Diseases Group (%)	*p* Value
Total	34 (38.63%)	54 (61.36%)	
Gender			
Female	28 (82.35%)	41 (74.54%)	*p* = 0.475 *
Male	6 (17.65%)	13 (23.64%)	
Age (mean)	66 years	54.88 years	
	SD ± 10.21	SD ± 15.3	
Range	27–83 years	23–86 years	
Level of education			
Primary	14 (41.17%)	16 (29.62%)	*p* = 0.193 *
Secondary	7 (20.58%)	21 (38.89%)	
Tertiary	13 (38.23%)	17 (31.48%)	
Smoking status			
Non-smoker	26 (76.47%)	39 (72.22%)	*p* = 0.659 *
Smoker and former smoker	8 (23.52%)	15 (27.77%)	
Environment			
Urban	31 (91.17%)	43 (79.62%)	*p* = 0.149 *
Rural	3 (8.82%)	11 (20.37%)	
Most frequent associated medical and surgical diseases			
Appendicectomy	5 (14.70%)	14 (25.92%)	
Cholecystectomy	6 (17.64%)	5 (9.25%)	
Diabetes mellitus	3 (8.82%)	4 (7.40%)	
Hypertension	6 (17.65%)	11 (20.37%)	
Ischemic heart disease	8 (23.52%)	7 (12.96%)	
Another autoimmune disease	10 (29.41%)	4 (7.40%)	
Duration of symptoms (months) mean ± SD	20.32 ± 41.04	6.07 ± 8.37	*p* = 0.015 **
Direct immunofluorescence findings			
IgG	29 (85.29%)	35 (64.81%)	
C3	17 (50%)	28 (51.85%)	
IgA	9 (26.47%)	11 (20.37%)	
Fibrinogen	9 (26.47%)	8 (14.81%)	
IgM	1 (2.94%)	8 (14.81%)	

* Chi-Square test, ** *t*-test.

**Table 3:** Location of oral lesions.

	Subepithelial Autoimmune Diseases Group No. (%)	Intraepithelial Autoimmune Diseases Group No. (%)	*p* Value
Gingiva	27 (79.41%)	15 (27.77%)	*p <* 0.00001 *
Tongue-dorsal surface	4 (11.76%)	5 (9.25%)	*p* = 0.729 **
Tongue-ventral surface	1 (2.94%)	11 (20.37%)	*p* = 0.252 *
Tongue-margins	0 (%)	4 (7.40%)	*p* = 0.155 **
Floor of the mouth	3 (8.82%)	5 (9.25%)	*p* = 0.999 **
Lips	1 (2.94%)	6 (11.11%)	*p* = 0.241 **
Labial mucosa	5 (14.70%)	9 (16.67%)	*p* = 0.999 **
Hard palate	6 (17.65%)	2 (3.70%)	*p =* 0.048 **
Soft palate	11 (32.35%)	25 (46.29%)	*p =* 0.032 *
Buccal mucosa	14 (41.17%)	45 (83.33%)	*p <* 0.0001 *
Retromolar region	1 (2.94%)	9 (16.67%)	*p* = 0.081 **
Tonsillar pillar	3 (8.82%)	15 (27.77%)	*p* = 0.055 **

* Chi-Square test; ** Fisher exact test.

## Data Availability

The data presented in this study are available on reasonable request from the corresponding author.

## Supplementary Materials

The following supporting information can be downloaded at: https://www.mdpi.com/article/10.3390/medicina60010167/s1.
